# Longitudinal Neuroanatomical Increases from Early to One-Year Postpartum

**DOI:** 10.21203/rs.3.rs-4432804/v1

**Published:** 2024-06-03

**Authors:** Alexander Dufford, Genevieve Patterson, Pilyoung Kim

**Affiliations:** Oregon Health & Science University; University of Denver; University of Denver

**Keywords:** postpartum, parenting, caregiving, subcortical, surface morphometry, longitudinal

## Abstract

Preclinical studies have provided causal evidence that the postpartum period involves regional neuroanatomical changes in ‘maternal’ brain regions to support the transition to offspring caregiving. Few studies, in humans, have examined neuroanatomical changes from early to one-year postpartum with longitudinal neuroimaging data and their association with postpartum mood changes. In this study, we examined longitudinal changes in surface morphometry (cortical thickness and surface area) in regions previously implicated in the transition to parenthood. We also examined longitudinal volumetric neuroanatomical changes in three subcortical regions of the maternal brain: the hippocampus, amygdala, and ventral diencephalon. Twenty-four participants underwent longitudinal structural magnetic resonance imaging at 2–4 weeks and 1 year postpartum. Cortical thickness increased from early to one-year postpartum in the left (*p* = .003, Bonferroni corrected) and right (*p* = .02, Bonferroni corrected) superior frontal gyrus. No significant increases (or decreases) were observed in these regions for surface area. Volumetric increases, across the postpartum period, were found in the left amygdala (*p* = .001, Bonferroni corrected) and right ventral diencephalon (*p* = .01, Bonferroni corrected). An exploratory analysis of depressive symptoms found reductions in depressive symptoms from early postpartum to one-year postpartum were associated with greater cortical thickness in the superior frontal gyrus for both the left (*p* = .02) and right (*p* = .02) hemispheres. The findings expand our evidence of the neuroanatomical changes that occur across the postpartum period in humans and motivate future studies to examine how mood changes across this period are associated with cortical thickness of the superior frontal gyrus.

## Introduction

Evidence from both animal and human studies suggest that neuroanatomical changes occur across the postpartum period to support the demands of parenthood.^1–5^ Several studies have used structural magnetic resonance imaging (MRI) to quantify these neuroanatomical changes; these studies have primarily focused on neuroanatomical changes across pregnancy and into the early postpartum period.^5–9^ However, there is evidence of long-lasting neuroanatomical changes occurring in postpartum period in response to the initiation and maintenance of the parent-offspring relationship.^5,7,10^ This evidence suggests that neuroanatomical changes across pregnancy may be hormonally driven and primarily result in *decreases* in brain structural metrics, while changes across the postpartum may be driven by and to support parent-offspring interactions via experience-dependent plasticity and result in *increases*.^3,5^ In the present study, we examined the longitudinal neuroanatomical changes from early to one-year postpartum using structural MRI. We focus on changes from early to one-year postpartum given the first year postpartum is when birthing parents are most vulnerable to psychopathology^11,12^ and it is during this time that long-term parent-child emotional bonds are established.^13,14^ Regarding postpartum mood, few studies that have examined perinatal longitudinal brain changes, especially in the postpartum, have examined how brain structure is associated with changes in mood symptoms across the postpartum period. Here, we conduct an exploratory analysis of the association between the change in depressive symptoms from early to one-year postpartum and brain structure at one-year postpartum.

Causal evidence from non-human animal studies has provided evidence that the transition to parenthood is marked by drastic and dynamic changes to brain structure to support parturition and the establishment and maintenance of parental behaviors.^1,15–18^ These studies have found that structural brain plasticity in the postpartum period occurs in specific brain regions involved in parental behaviors. In the early-to-mid postpartum period (PD1 to PD14, roughly equivalent to one-year postpartum), increased dendritic spine density has been observed in the hippocampus,^19,20^ medial prefrontal cortex,^21^ nucleus accumbens,^22^ and cingulate cortex.^23^ During the early and mid-postpartum period, studies have found increases in dendritic length and arborization in the medial prefrontal cortex,^21^ nucleus accumbens,^24^ and medial preoptic area of the hypothalamus.^24^ In terms of grey matter changes, a longitudinal MRI study of dams found grey matter increases in the medial preoptic area, paraventricular hypothalamic nucleus, and amygdala.^4^ Across the structural metrics used in the animal studies of the postpartum period, consistent evidence suggests regional structural *increases* from early to late postpartum.

Human studies of the neuroanatomical changes across the perinatal period have examined both gray matter volume and surface morphometry metrics (primarily cortical thickness and surface area) from structural MRI.^3^ Gray matter volume is the product of cortical thickness (CT) and surface area (SA)^25^; however, it is critical to examine CT and SA in addition to gray matter volume as these measures have unique developmental trajectories,^26^ associations with behavior,^27^ and environmental/genetic contributions.^28,29^ Motivated by animal studies, human studies have begun to examine CT in the postpartum period.^3,30^ While it was a cross-sectional study, we previously found a correlation between postpartum timing and CT for primiparous individuals.^30^ Individuals further into the further into the first 6 months of the postpartum period had greater CT in the superior frontal, caudal middle frontal, lateral occipital, and precentral gyri. These findings of greater CT in the postpartum are not incompatible with several studies finding regional cortical thickness decreases of the medial frontal cortex, precuneus, posterior cingulate, inferior frontal gyri, and superior temporal sulci during pregnancy as regions have little anatomical overlap and the underlying processes for pregnancy-related decreases and postpartum-related increase are hypothesized to be unique^3,31^.

Regarding longitudinal brain volumetric changes in the postpartum period in humans, gray matter volume increases have been reported globally,^32,33^ regionally,^34,35^ and locally.^2,36^ In one study, gray matter volume at 2–4 weeks was compared to volume at 3–4 months postpartum. Longitudinal gray matter volume increases were observed in the hypothalamus, substantia nigra, and globus pallidus in addition to large regions of the prefrontal cortex and parietal lobes.^2^ The increases in the hypothalamus, substantia nigra, and amygdala were associated with positive perceptions of the participant’s infant. Luders et al., found voxel-wise gray matter volume increases from 1–2 days postpartum to 4–6 week postpartum. The regions found were widespread and included both cortical (pre- and postcentral gyri, inferior frontal gyrus, and frontal operculum) and subcortical (thalamus and caudate) regions.^33^ Using the same dataset, longitudinal increases were found for gray matter volume in the amygdala, particular in the superficial subarea of the amygdala.^37^ Across these studies, neuroanatomical changes have been consistently reported in the postpartum period for the hippocampus, amygdala, and hypothalamus.

Non-human animal studies have also provided evidence of associations between depressive-like behaviors and brain structural changes across the postpartum.^5,15,22,38^ Understanding the associations between postpartum mood and postpartum brain changes is critical as one out of eight individuals experience elevated depressive symptoms in the postpartum period.^39^ However, in humans, the neurobiological mechanisms underlying postpartum depression remain elusive. Regarding associations between mood symptoms and neuroanatomical change in the postpartum period, one study found that increases in the right superficial subregion of the amygdala from 1–2 days after childbirth to 4–6 weeks after childbirth, were associated with decreases in state anxiety symptoms.^37^ This study provides some preliminary evidence of associations between postpartum neuroanatomical changes and postpartum mood and, but if changes in mood symptoms, particularly depressive symptoms, across the postpartum are prospectively associated with postpartum brain structure is unclear. Examining these associations is critical as postpartum depressive symptoms may impact the neuroanatomical changes that support parenting as evidence suggests higher postpartum depressive symptoms are associated with reduced emotion regulation of the parent^40,41^ and lower parental sensitivity.^42,43^

The present study aims to further characterize neuroanatomical changes (for both surface morphometry and gray matter volume) across the postpartum period. To expand on previous longitudinal neuroimaging studies, we conducted the first MRI session in the early postpartum period (2–4 weeks postpartum, Early Postpartum) and approximately one-year later (One-Year Postpartum). For Kim et al., the second scan timepoint was 4–6 weeks postpartum, therefore longitudinal neuroanatomical change across the first year postpartum is unclear. Further, few studies have examined longitudinal changes in CT and SA from early to one-year postpartum. Based upon the regions in which the CT of primiparous mothers was associated with postpartum months for the first 8 months in the previous cross-sectional study,^30^ we hypothesized longitudinal increases of CT from early to one-year postpartum in the superior frontal gyrus, lateral occipital gyrus, caudal middle frontal gyrus, and precentral gyrus. As SA across the postpartum period is unclear, we analyzed SA only in the regions examined for the CT analysis. Further, we conducted a post-hoc test in which we examined the neuroanatomical change in regions that have been previously found to decrease in CT across pregnancy.^7^

Regarding subcortical volume, we hypothesized, based upon the previous human and animal studies,^3,5,15,16^ that gray matter volume of the hippocampus, amygdala, and ventral diencephalon will increase from the early to one-year postpartum period. The analysis focused on the gray matter volume of the ventral diencephalon as longitudinal automatic segmentations of the hypothalamus have yet to be developed^44^ However, Freesurfer’s ‘recon-all’ pipeline provides a segmentation referred to as the ventral diencephalon which includes the hypothalamus with mamillary body, subthalamic, lateral geniculate, medial geniculate and red nuclei, substantia nigra and the surrounding white matter.^45^ Further, we conducted a post-hoc test in which examined the regions that have been previously found to decrease in CT across pregnancy^9^. Lastly, we conducted an exploratory analysis to examine if changes in depressive symptoms from early to one-year postpartum were associated with brain structural metrics (CT for cortical regions and volume for subcortical regions) at one-year postpartum.

## Materials and Methods

### Participants.

Participants were recruited during pregnancy from the Department of Obstetrics and Gynecology at hospitals in the Denver metro area. Eligibility criteria were: 1) over 18 years of age; 2) singleton intrauterine pregnancy; 3) prior to 16 weeks of gestation; 4) fluency in English; and 5) a family income-to-needs ratio below 8 (based upon income information gathered from an initial phone screening with participants). Exclusion criteria include: 1) current psychotropic medication use; 2) current or lifetime psychiatric/neurological illness other than depression and anxiety diagnosis; 3) maternal substance use except for occasional use of alcohol, cigarettes, or cannabis (assessed using maternal reports and urine toxicology); 4) obstetric risk conditions such as systemic maternal disease, placental or cord abnormalities, uterine anomalies, infection, chromosomal abnormalities; 5) corticosteroid medication usage during their pregnancy; or 6) nonremovable ferromagnetic metal in or on the body (for safety in the magnetic resonance imaging MRI scanner). As recruitment aimed at a representative community sample, individuals with a history of depression and/or anxiety disorders were included as these are the two most common mental disorder diagnoses of the perinatal period.^46–48^ As this data was taken from a larger study focused on stress exposure in the postpartum period, the sample was primarily comprised of low- and middle-income participants and excluded participants that were currently experiencing high-income.

### Procedures.

Data for the present study was taken from a larger study of examining stress exposure across the perinatal period. Participants in the study participated in 6 research visits across the perinatal period, with 4 home visits (12–16, 22, 32 weeks of pregnancy, and 2–4 weeks postpartum) and 2 neuroimaging visits (denoted at Early Postpartum and One-Year Postpartum) at 2–4 weeks postpartum and approximately one-year postpartum. At the first home visit, participants were given information about the study and provide informed consent. The neuroimaging visits occurred at the Center for Innovation & Creativity at the University of Colorado – Boulder. Childcare support was provided to each family in addition to monetary compensation for the participant’s time at the end of each visit. All procedures were approved by the University of Denver Institutional Review Board.

### Edinburgh Postnatal Depression Scale (EPDS).

The EPDS^39^ was administered to participants twice, once at the home visit occurring at 2–4 weeks postpartum and again at the home visit occurring at approximately one-year postpartum. The EPDS is a 10-item screening questionnaire used to examine postpartum depressive symptoms in both research and clinical settings. For each item, the respondent chooses from a range from 0 “not at all” to 3 “yes, most of the time/as much as I always could”. Higher total scores indicated higher depressive symptoms an scores above 9 may indicate ‘elevated’ depressive symptoms.^49,50^ Three participants were missing EPDS data for the study, this reduced the sample size for the exploratory analysis to 21 participants.

### Parity.

There is evidence from animal studies of associations between parity and brain structure in the postpartum period.^31^ Studies in humans have also found associations between parity and ‘brain age’ which measures the aging levels of an individual’s brain in comparison to their peers.^51,52^ Therefore, upon study entry, parity was measured by asking each participant how many live births they have had before the current pregnancy.

### Anatomical MRI Acquisition.

All MRI data were collected using a Siemens 3T MAGNETOM Prisma scanner with a 32-channel head coil. High-resolution, T1-weighted structural images (3D magnetization-prepared rapid acquisition gradient-recalled echo sequence, MP-RAGE) were acquired for both timepoints (early and one-year postpartum) with the following parameters: TR = 2400 ms, TE = 2.22 ms, inversion time = 1000 ms, voxel size = 0.8 × 0.8 × 0.8 mm.

### Anatomical MRI Quality Control Procedure.

The anatomical MRI quality control procedure (QC) used a combined approach that involved both visual and image-derived QC steps. First, each T1-weighted image was visually inspected, slice-by-slice, and assigned a rating from 1 to 4 based upon its overall quality.^53^ Based on the visual QC step, all images passed and were included in the image-derived QC procedure. For the image-derived QC, T1-weighted images were processed with the anatomical workflow in MRIQC.^54^ This workflow calculates image quality metrics (IQMs) at the participant level and provides group-level summaries and plots of the IQMs across the sample. Based upon both image coefficient of join variation (cjv)^55^ and contrast-to-noise ratio (cnr)^56^, no anatomical image was determined to be an outlier in terms of these metrics.

### Anatomical MRI Analysis.

Results included in this manuscript come from preprocessing performed using sMRIPprep 0.12.0^57^ (RRID:SCR_016216), which is based on Nipype 1.8.6.^58^

### Cross-sectional Anatomical Data Preprocessing.

A total of 1 T1-weighted (T1w) image was found within the input BIDS dataset per timepoint. Each T1-weighted (T1w) image was corrected for intensity non-uniformity (INU) with N4BiasFieldCorrection,^59^ distributed with ANTs 2.3.3,^60^ and used as T1w-reference throughout the workflow. The T1w-reference was then skull-stripped with a Nipype implementation of the antsBrainExtraction.sh workflow (from ANTs), using OASIS30ANTs as target template. Brain tissue segmentation of cerebrospinal fluid (CSF), white-matter (WM) and gray-matter (GM) was performed on the brain-extracted T1w using fast (FSL 6.0.5.1:57b01774, RRID:SCR_002823).^61^ Brain surfaces were reconstructed using recon-all (FreeSurfer 7.3.2, RRID:SCR_001847)^62^, and the brain mask estimated previously was refined with a custom variation of the method to reconcile ANTs-derived and FreeSurfer-derived segmentations of the cortical gray-matter of Mindboggle (RRID:SCR_002438)^63^.

For more details of the pipeline, see the section corresponding to workflows in sMRIPrep’s documentation. After each image was processed with sMRIPrep, visual inspection of the segmentations and surface reconstructions were conducted following previously developed procedures.^64^

### Longitudinal Anatomical Data Preprocessing.

After each individual session was processed with recon-all cross-sectionally, we used Freesurfer-7.3.2’s longitudinal processing pipeline.^65^ This pipeline has been extensively described elsewhere.^65^ Briefly, to facilitate unbiased longitudinal data analysis of anatomical data, the processing pipeline involves three steps. First, we processed the anatomical data cross sectionally. For the second step, the pipeline creates a template or ‘base’ for each participant from all their timepoints and estimates the average anatomy (also referred to as the within-subject template). An unbiased median image is used as the template and segmentation and surface reconstruction were performed. In the third step, we process the scans ‘longitudinally’ in which information from the within-subject template and from each of the time points is used to initialize the algorithms used for recon-all. For the subcortical segmentation, the pipeline creates a fused segmentation for each time point using an intensity-based probabilistic voting scheme. To QC the longitudinal processing steps, we conducted visual QC of tissue segmentation and subcortical segmentation accuracy by overlaying the segmentations on the T1 image for the within-subject template (base).

As with the cross-sectional processing, all segmentations passed QC procedures. To conduct the region of interest analysis, subcortical segmentation volumes (from the ‘aseg’ output) were compiled for the bilateral hippocampus, bilateral amygdala, and bilateral ventral diencephalon (using the asegstats2table command). Regional mean cortical thickness and surface area values (from the ‘aparc’ output) were also compiled for the statistical analysis (using the aparcstats2table command). Analysis focused on the Desikan-Killiany Atlas parcellation in Freesurfer to be in alignment with the regions analyzed in Kim, Dufford, & Tribble, 2016.

### Linear Mixed Effects Models.

To examine the longitudinal change of the regions of interest, we used Linear Mixed Effects modeling implemented by the “lme4”^66,67^ built under R version 4.2.3. We tested random intercept models for each region of interest. For each of these models, the scan timepoint, age at the early postpartum scan, parity, and global structural measure (for CT it is appropriate to covary for global effects using mean thickness for the hemisphere, for SA the sum SA for the hemisphere, and for volume, the total intracranial volume for both hemispheres) were included as fixed effects. For each model ‘Participant’ was modelled as a random effect. For each model *p*-values were computed using the “lmerTest”^68,69^ package in R as these evaluations of significance have been shown to have Type I error rates closest to *p* = .05.^70^ Uncorrected *p*-values were corrected for multiple testing using Bonferroni correction implemented in the R function “p.adjust”. We also conducted a post-hoc test, using identical models as described above, but tested regions of interest (ROI) from a previous study^9^ that have found pregnancy-related decreases in structural volume and thickness including the fusiform gyrus, inferior frontal gyrus, precuneus, superior temporal gyrus. These results are presented in the **Supplementary Information**.

### Exploratory Analysis of Depressive Symptoms.

For the exploratory analysis, we used Pearson correlations to examine the associations between the change in depressive symptoms from early to one-year and CT at one-year postpartum. We focused this analysis to only regions that had a significant change from early postpartum to one-year postpartum from the ROIs. Identical exploratory tests were also tested for the left amygdala and right ventral diencephalon.

## Results

### Demographic Variables.

Demographic information for the sample is presented in Table 1. A correlation table of the study variables is presented in **Table 2**. For the EPDS measured in the early postpartum, 4 participants had a total score >10, a commonly used cutoff score indicating ‘mild depression’.^71^ For the one-year postpartum scan, 3 participants had a total score > 10. The mean change in depressive symptoms (EPDS) from early to one-year postpartum was 1.19 (3.59) suggesting that on average, depressive symptoms increased slightly from early to one-year postpartum.

### Linear Mixed Effects Models for Surface Morphometry.

**Figure 1:**
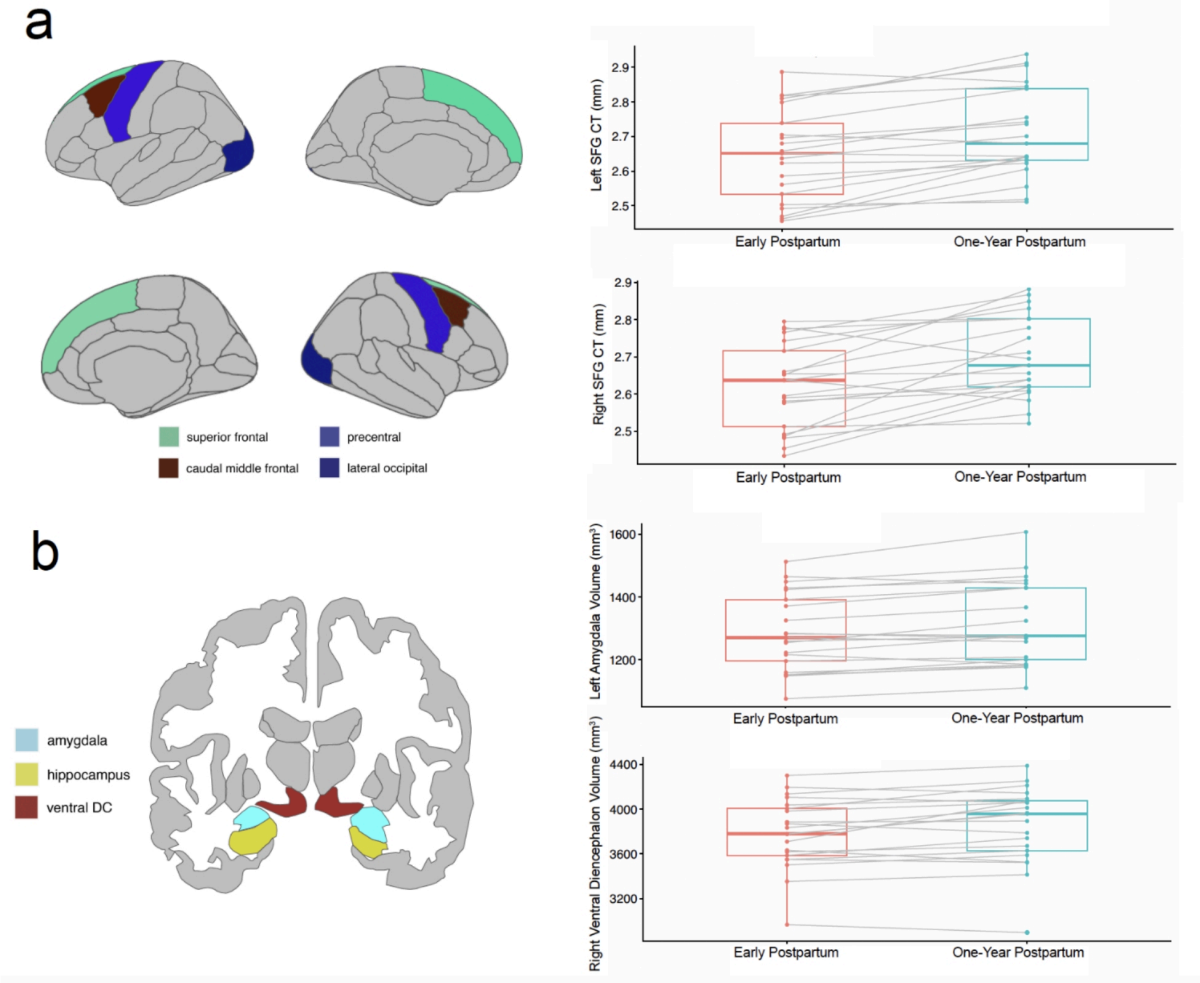
(a) Regions of interest for the cortical thickness (CT) analysis. Plots show the changes in CT for the superior frontal gyrus (SFG) from early to one-year postpartum. (b) Regions of interest for the subcortical volume analysis. Plots show the changes in the left amygdala and ventral diencephalon from early to one-year postpartum.

We fitted a linear mixed model (estimated using REML and nloptwrap optimizer) for left superior frontal gyrus CT with scan timepoint (Early Postpartum or One-Year Postpartum, age at the early postpartum scan, parity, and mean cortical thickness of the left hemisphere (formula: left superior frontal gyrus cortical thickness ~ 1 + scan timepoint + age at the early postpartum scan + parity + mean cortical thickness of the left hemisphere). The model included participant as random effect (formula: ~1 | Participant). The model’s total explanatory power is substantial (conditional *R*^*2*^ =0.89) and the part related to the fixed effects alone (marginal *R*^*2*^) is of 0.45. The model’s intercept was nonsignificant: 0.64 (95% CI [−0.18, 1.47], *t*(41) = 1.57, *p* = 0.123). For this model, the effect of scan timepoint was statistically significant and positive (beta = 0.10, 95% CI [0.05, 0.15], *t*(41) = 4.14, *p* < .001; Std. beta = 0.03, 95% CI [0.01, 0.04], see Figure 1 and Table 3). An identical model was fit using the right superior frontal gyrus values and mean cortical thickness of the right hemisphere as a covariate (in addition to scan timepoint, age at the early postpartum scan, parity). This model’s total explanatory power is substantial (conditional *R*^*2*^ = 0.89) and the part related to the fixed effects alone (marginal *R*^*2*^) is of 0.69. The model’s intercept was nonsignificant at 0.10 (95% CI [−0.63, 0.82], *t*(41) = 0.27, *p* = 0.790). Within this model, the effect of scan timepoint was statistically significant and positive (beta = 0.04, 95% CI [0.01, 0.06], *t*(41) = 3.16, *p* = 0.003; Std. beta = 0.02, 95% CI [7.19e-03, 0.03]). Model results for the regions of interest are presented in **the Supplemental Information**.

### Linear Mixed Effects Models for Subcortical Volumes.

A linear mixed model was t for the left amygdala and right ventral diencephalon. For the left amygdala, the model’s conditional *R*^*2*^ = 0.98) and marginal R^*2*^ was of 0.07. The model’s intercept was significant at 1086.01 (95% CI [418.07, 1753.94], *t*(41) = 3.28, *p* = 0.002). The effect of scan timepoint was statistically significant and positive (beta = 25.80, 95% CI [13.53, 38.07], *t*(41) = 4.25, *p* < .001; Std. beta = 0.10, 95% CI [0.05, 0.15], see Figure 1 and **Supplementary Table 5**). For the right diencephalon, the conditional *R*^*2*^ was 0.95 with a marginal *R*^*2*^ of 0.26. The model’s intercept was significant: 1951.54 (95% CI [456.32, 3446.76], *t*(41) = 2.64, *p* = 0.012). The effect of scan timepoint was statistically significant and positive (beta = 73.85, 95% CI [29.00, 118.69], *t*(41) = 3.33, *p* = 0.002; Std. beta = 0.12, 95% CI [0.05, 0.19], see Figure 1 and **Supplementary Table 6**). Results for the hippocampus that were not significant are presented in **Supplementary Table 4**.

As they were separate structural metric analyses, Bonferroni correction was conducted across all eight CT regions (left and right superior frontal gyrus, left and right caudal middle frontal gyrus, left and right lateral occipital gyrus, left and right precentral gyrus), all eight SA regions (same regions as CT), and six subcortical volume regions (left hippocampus, right hippocampus, left amygdala, right amygdala, left ventral diencephalon, right diencephalon) separately. After Bonferroni correction, longitudinal increases were found for the left (*p* = .003, corrected) and right (*p* = .02, corrected) superior frontal gyrus CT. There was no significant increase or decrease for the regions of interest for SA (*ps* > .05, corrected). After Bonferroni correction, significant increase in gray matter volume was observed for the left amygdala (*p* = .001, corrected) and right ventral diencephalon (*p* = .01, corrected).

### Exploratory Analyses of Depressive Symptoms.

**Figure 2:**
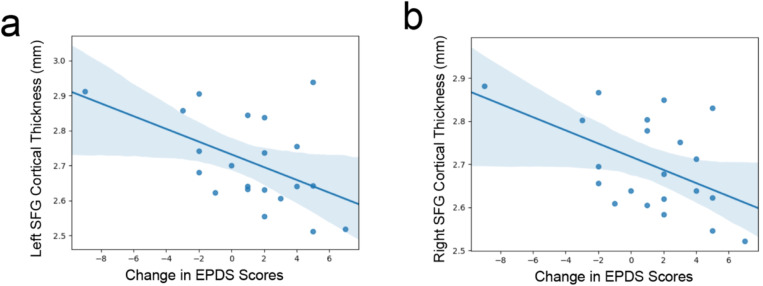
Scatter plots for the exploratory analyses examining change in depressive symptoms from early to one-year postpartum and left (a) and right (b) superior frontal gyrus thickness.

For the exploratory analyses we examined the associations between longitudinal change in depressive symptoms and brain structure. First, we determined if there were any statistical outliers in the change in depressive symptoms by examining the *z*-scored values. The participant that had a change in EPDS score of ‘−8’ was not considered an outlier as it had a *z*-score value of −2.84, and −3 to 3 is the traditionally used cutoff.^72^ The correlation between change in depressive symptoms (from early to one-year postpartum) for left superior frontal gyrus CT was negative and statistically significant (*r* = −0.50, 95% CI [−0.77, −0.09], *t*(19) = −2.54, *p* = 0.020, see Figure 1). Again, a similar pattern was observed for the association between changes in depressive symptoms and right superior frontal gyrus CT (*r* = −0.50, 95% CI [−0.77, −0.09], *t*(19) = −2.54, *p* = 0.020, see Figure 2). However, if the participant who’s change in symptom score that had a *z*-score of −2.84 was removed from the analysis, these correlations were no longer significant and at a trend level (*p* = .09 for the left superior frontal gyrus and *p* = .11 for the right superior frontal gyrus). All correlations between left amygdala volume, right ventral diencephalon volume, and depressive symptoms (and depressive symptom change) were nonsignificant (*ps* > .05).

## Discussion

Human neuroimaging has provided a lens to understand the neuroanatomical changes that occur across the perinatal period.^3,5,16,31^ Understanding these brain changes is critical to further our understanding of the neural underpinnings of the onset and maintenance of parental behaviors. The present findings expand upon our knowledge of postpartum brain changes by quantifying structural changes from the early postpartum period to one-year postpartum. Further, we examined both CT and subcortical volumes and found significant increases in bilateral superior frontal gyrus CT, left amygdala volume, and right ventral diencephalon volume. The surface morphometry analysis focused on four regions of interest that we previously found CT was associated with postpartum timing.^30^ Of these four regions, longitudinal neuroanatomical increases in CT were found for the left and right superior frontal gyrus. As the association between postpartum brain structure and maternal mood is unclear, we conducted an exploratory analysis in which we tested the correlation between the regions that significantly increased in the postpartum and depressive symptoms in the postpartum period. In an exploratory analysis, we found that reductions of depressive symptoms from early postpartum to one-year postpartum were associated with greater bilateral superior frontal gyrus CT at one-year postpartum. We provide evidence for future studies to examine and attempt to replicate the association between changes in depressive symptoms across the postpartum period and superior frontal gyrus CT correlation with using a larger, clinically-enriched sample.

The present study was motivated by our previous study that found positive associations (and no negative associations) between postpartum timing and CT in the super frontal, caudal middle frontal, lateral occipital, and precentral gyri.^30^ As this study was cross-sectional, we could not examine if the CT in these regions ‘changed’ across the postpartum. Here, we found that of the regions correlated with postpartum timing in our previous study, only the superior frontal gyrus had significant increases in CT from early postpartum to one-year postpartum. This finding is aligned with other studies that have found structural increases in the regions of the prefrontal cortex overlapping with the superior frontal gyrus. The mechanism of these structural increase in the prefrontal cortex remains unclear, although it is hypothesized, they are due to experience-dependent plasticity.^5,30,31^ This is hypothesized to include responding to the increased regulatory demands of parenting,^1,5^ affective regulation for both parent and child,^5,73^ and needing to recognize and respond appropriately/flexibly to the offspring’s needs.^74^ Evidence from animal studies support these hypotheses and have found increased neuronal spine density from the early to late postpartum^21^ as well as postweaning.^74^ If these neuronal increases underlie the increases observed in the CT increases observed with structural MRI remains to be confirmed. The superior frontal gyrus is a large region of cortex, therefore more precise localization of these increases is required with larger studies that can utilize vertex-wise analytic methods.

For the subcortical regions of interest, we have replicated previous studies that have found structural increases in amygdala volume across the postpartum period.^2,33,37^ It has been found in a previous study that amygdala volume increases from 1–2 days after childbirth to 4–6 weeks after childbirth.^33^ These findings were further examined in terms of amygdala subareas in which the most pronounced increases were found in the superificial region.^37^ Further, amygdala volume was also found to increase, using a voxel-wise approach, from 2–4 weeks to 3–4 months postpartum.^2^ We extend this knowledge by replicating this finding from 2–4 weeks to one-year postpartum. The amygdala is recognized to be a core region of the ‘maternal’ brain for its role in salience detection,^1,73,75^ parent-child interaction,^76^ and affective processing.^75^ Regarding potential neuronal mechanisms for the observed increases in the amygdala, animal studies suggest that dendritic spine concentrations increase post-parturition in the anterodorsal medial amygdala.^77^ In addition to the amygdala, we replicate findings of postpartum structural increases in regions that include the hypothalamus.^2^ We found structural increases of the ventral diencephalon which includes the hypothalamus, mamillary bodies, subthalamic, lateral geniculate, medial geniculate and red nuclei, substantia nigra and surrounding white matter.^45^ Therefore, future studies will need to use recent advances in image segmentation for the smaller structures included in the ventral diencephalon segmentation (such as the mamillary bodies) as they have been implicated to be involved in infant caregiving.^78^ Specifically, neuronal dendritic branching of the medial preoptic area of the hypothalamus has been found to increase across the postpartum period in animal studies and impact the onset of parenting behaviors.^24,79,80^

We did not find evidence of significant increase in gray matter volume for the hippocampus. However, this may be due to lack of power given a significant *p*-value for the left hippocampus before multiple comparisons correction. Animal studies indicate decreases in neuronal proliferation in the middle and late postpartum periods.^81,82^ Studies have also found dendritic remodeling across the perinatal period for the CA1 and CA3 regions of the hippocampus.^83^ In a small sample of humans (n = 11) pregnancy-related decreases had returned to pre-pregnancy baseline except for in the left hippocampus, which had a partial recovery.^9^ Therefore, future studies with pre-pregnancy, prenatal, and postnatal scanning may be needed to examine the complex trajectories of hippocampal structure across the perinatal period.

A major strength of the present study was the ability to examine if change in depressive symptoms from early to one-year postpartum were associated with brain structure at one-year postpartum. As several animal studies have provided causal evidence of postpartum structural changes impacting depressive-like behaviors,^22^ we conducted an exploratory analysis to examine if changes in depressive symptoms across the postpartum were associated with brain structural metrics. We found reductions in depressive symptoms from early to one-year postpartum were associated with greater CT in both the left and right superior frontal gyrus. Previous studies have implicated the superior frontal gyrus in studies of participants diagnosed with postpartum depression including a positive correlation observed with EPDS scores^84^ and reduction of functional connectivity between the anterior cingulate cortex and bilateral superior frontal gyrus.^85^ As mentioned, more precise localization of these potential brain-symptom correlations is needed given the large anatomical region defined as the superior frontal gyrus. Other studies of perinatal brain changes have not found associations between brain structure and depressive symptoms.^5^ This may be due to several factors. First, few studies have examined mood symptoms longitudinally in the postpartum period and therefore could not examine changes in symptoms. Further, it is worth noting the present sample, in comparison to previous perinatal neuroimaging studies, is diverse in terms of the socioeconomic and ethnic backgrounds of the participants.

The findings from the present study should be interpreted considering the following limitations. First, the sample size was modest for longitudinal neuroimaging and will need replication in larger samples. The modest sample size motivated the analytic approach of focusing on regions of interest found in previous studies, rather than mass-univariate approaches (vertex-wise or voxel-wise). However, other studies using a region of interest approach have found effects at similar sample sizes such as 14^37^ and 19^2^. Future studies, with larger samples, will be able to harness advances in more computationally efficient linear mixed modeling approaches for neuroimaging data.^86^ Second, the ventral diencephalon is comprised of multiple unique brain regions and with the segmentation that was used, we could not delineate which structures were contributing most to the longitudinal increase in volume. Future studies will use deep-learning based segmentations of these regions and examine specific hypothalamic subunits.^44^ Third, the modest sample size resulted in the brain-behavior correlation being conducted as exploratory. Relatedly, the findings of the superior frontal gyrus correlation with depressive symptom change were found in a community-based sample, this relation will need replication in a clinically-enriched sample with higher levels of depressive symptoms. Last, the use of dense longitudinally sampling^87–90^ across the perinatal transition is still critical to establish prenatal to postnatal trajectories for brain structure.

## Conclusions

Despite the troubling lack of neuroimaging studies focusing on women’s health issues, consistency is being found across longitudinal neuroimaging studies of the perinatal period.^3^ Here, we provide additional support for the hypothesis that the postpartum period entails increase in brain structural metrics. Specifically, we found increases from the early postpartum to one-year postpartum in the bilateral superior frontal gyrus in terms of CT, and no significant decreases. We did not find any increases or decreases in cortical SA for the regions of interest (the superior frontal, caudal middle frontal, lateral occipital, and precentral gyri). For subcortical gray matter volume, longitudinal increases were found for the left amygdala and right ventral diencephalon. Lastly, we found that reductions in depressive scores from early postpartum to one-year postpartum were associated with greater thickness of the bilateral superior frontal gyrus. Here, we add evidence to the emerging understanding of the longitudinal neuroanatomical changes across the perinatal period by focusing on the early postpartum to one-year postpartum. Further, we find exploratory evidence that changes in depressive symptoms across the postpartum period may play a role in the neuroanatomical increased observed from early to one-year postpartum. This finding can provide a foundation for future longitudinal studies of the postpartum period to further identify and/or replicate brain regions that may confer risk and resilience to changes in postpartum mood.

## Figures and Tables

**Table 1.** Demographic information for the sample (EPDS = Edinburgh Postnatal Depression Scale).

Variable	Sample (N=24)
Maternal Age at Early Postpartum Scan (years)	28.04 (4.92)
Maternal Age at One-Year Postpartum Scan (years)	29.08 (4.88)
Maternal Education (years)	14.83 (2.79)
Maternal Income-to-needs Ratio	2.94 (2.52)
Maternal Ethnicity (Hispanic)	11 (47%)
Maternal Race	
Black/African American	2 (8%)
White/Caucasian	12 (52%)
American Indian/Alaska Native	3 (13%)
Other	6 (26%)
Number of Live Births Upon Study Entry	1.16 (1.55)
Interval Between Scans (months)	15.31 (5.5)
EPDS Total Score at Early Postpartum Scan	5.52 (4.15)
EPDS Total Score at One-Year Postpartum Scan	6.71 (4.69)
Change in EPDS from Early to One Year Postpartum	1.19 (3.59)

Note. N=3 missing EPDS Total Score at Early Postpartum Scan. N=3 missing EPDS Total Score at One-Year Postpartum Scan. N=3 missing Change in EPDS from Early to One Year Postpartum. N=1 missing Maternal Education (years). N=2 missing Maternal Income-to-needs Ratio. N=1 missing Maternal Ethnicity (Hispanic). N=1 missing Maternal Race,

**Table 3.** Results from the linear mixed models for superior frontal gyrus cortical thickness. *P*-value corr. is the Bonferroni corrected *p*-value.

	Left Superior Frontal Gyrus CT			Right Superior Frontal Gyrus CT		
*Predictors*	*Estimates*	*CI*	*p (p value corr.)*	*Estimates*	*CI*	*p (p value corr.)*
(Intercept)	0.69	−0.14 – 1.52	0.1	0.1	−0.63 – 0.82	0.79
Scan Timepoint	0.05	0.03 – 0.08	<0.001, 0.003	0.04	0.01 – 0.06	0.003, 0.02
Maternal Age at the Early Postpartum Scan	0	−0.01 – 0.00	0.367	0	−0.01 – 0.00	0.752
Live Births Upon Study Entry	0.01	−0.02 – 0.03	0.478	0.01	−0.01 – 0.02	0.405
Mean Thickness	0.85	0.52 – 1.17	<0.001	1.07	0.79 – 1.35	<0.001
Random Effects						
σ^2^	0	0				
τ_00_	0.01_subject_	0.00_subject_				
ICC	0.79	0.64				
N	24_subject_	24_subject_				
Observations	48	48				
Marginal R^2^ / Conditional R^2^	0.448 / 0.886	0.689 / 0.887				

## Data Availability

The data that support the findings of this study are available from the senior author upon reasonable request.

## References

[1] Kim P, Strathearn L, Swain JE (2016) The maternal brain and its plasticity in humans. Horm Behav 77:113–123 26268151 10.1016/j.yhbeh.2015.08.001 PMC4724473

[2] Kim P (2010) The plasticity of human maternal brain: longitudinal changes in brain anatomy during the early postpartum period. Behav Neurosci 124:695 20939669 10.1037/a0020884 PMC4318549

[3] Luders E, Kurth F, Poromaa IS (2022) The neuroanatomy of pregnancy and postpartum. NeuroImage 263:119646 36155243 10.1016/j.neuroimage.2022.119646

[4] Barrière DA (2021) Brain orchestration of pregnancy and maternal behavior in mice: A longitudinal morphometric study. NeuroImage 230:117776 33516895 10.1016/j.neuroimage.2021.117776

[5] Barba-Müller E, Craddock S, Carmona S, Hoekzema E (2019) Brain plasticity in pregnancy and the postpartum period: links to maternal caregiving and mental health. Arch Women Ment Health 22:289–299 10.1007/s00737-018-0889-z PMC6440938 30008085

[6] Paternina-Die M (2024) Women’s neuroplasticity during gestation, childbirth and postpartum. Nat Neurosci, 1–9 38182834 10.1038/s41593-023-01513-2 PMC10849958

[7] Hoekzema E (2022) Mapping the effects of pregnancy on resting state brain activity, white matter microstructure, neural metabolite concentrations and grey matter architecture. Nat Commun 13:6931 36414622 10.1038/s41467-022-33884-8 PMC9681770

[8] Carmona S (2019) Pregnancy and adolescence entail similar neuroanatomical adaptations: A comparative analysis of cerebral morphometric changes. Hum Brain Mapp 40:2143–2152 30663172 10.1002/hbm.24513 PMC6865685

[9] Hoekzema E (2017) Pregnancy leads to long-lasting changes in human brain structure. Nat Neurosci 20:287–296 27991897 10.1038/nn.4458

[10] Orchard ER (2020) Relationship between parenthood and cortical thickness in late adulthood. PLoS ONE 15:e0236031 32722686 10.1371/journal.pone.0236031 PMC7386609

[11] Serretti A, Olgiati P, Colombo C (2006) In uence of postpartum onset on the course of mood disorders. BMC Psychiatry 6:1–7 16438725 10.1186/1471-244X-6-4 PMC1373619

[12] Di Florio A (2014) Mood disorders and parity–a clue to the aetiology of the postpartum trigger. J Affect Disord 152:334–339 24446553 10.1016/j.jad.2013.09.034 PMC4025607

[13] Takács L, Smolík F, Kaźmierczak M, Putnam SP (2020) Early infant temperament shapes the nature of mother-infant bonding in the first postpartum year. Infant Behav Dev 58:101428 32135403 10.1016/j.infbeh.2020.101428

[14] Kinsey CB, Baptiste-Roberts K, Zhu J, Kjerulff KH (2014) Birth-related, psychosocial, and emotional correlates of positive maternal–infant bonding in a cohort of first-time mothers. Midwifery 30:e188–e194 24650812 10.1016/j.midw.2014.02.006 PMC4010321

[15] Servin-Barthet C (2023) The transition to motherhood: linking hormones, brain and behaviour. Nat Rev Neurosci 24:605–619 37612425 10.1038/s41583-023-00733-6

[16] Pawluski JL, Hoekzema E, Leuner B, Lonstein JS (2022) Less can be more: Fine tuning the maternal brain. Neurosci Biobehavioral Reviews 133:104475 10.1016/j.neubiorev.2021.11.045 PMC8807930 34864004

[17] Leuner B, Glasper ER, Gould E (2010) Parenting and plasticity. Trends Neurosci 33:465–473 20832872 10.1016/j.tins.2010.07.003 PMC3076301

[18] Lonstein JS, Pereira M, Morrell JI, Marler CA (2015) Parenting behavior. Physiol Reproduction: New York, 2371–2437

[19] Kinsley CH (2006) Motherhood and the hormones of pregnancy modify concentrations of hippocampal neuronal dendritic spines. Horm Behav 49:131–142 16005000 10.1016/j.yhbeh.2005.05.017

[20] Chen J-R (2017) Reproductive experience modified dendritic spines on cortical pyramidal neurons to enhance sensory perception and spatial learning in rats. Exp Anim 66:61–74 27784858 10.1538/expanim.16-0061 PMC5301002

[21] Hillerer KM (2018) Gating of the neuroendocrine stress responses by stressor salience in early lactating female rats is independent of infralimbic cortex activation and plasticity. Stress 21:217–228 29397787 10.1080/10253890.2018.1434618

[22] Haim A, Sherer M, Leuner B (2014) Gestational stress induces persistent depressive-like behavior and structural modifications within the postpartum nucleus accumbens. Eur J Neurosci 40:3766–3773 25359225 10.1111/ejn.12752 PMC4488909

[23] Salmaso N, Quinlan M, Brake W, Woodside B (2011) Changes in dendritic spine density on layer 2/3 pyramidal cells within the cingulate cortex of late pregnant and postpartum rats. Horm Behav 60:65–71 21397603 10.1016/j.yhbeh.2011.03.002

[24] Shams S (2012) Dendritic morphology in the striatum and hypothalamus differentially exhibits experience-dependent changes in response to maternal care and early social isolation. Behav Brain Res 233:79–89 22569575 10.1016/j.bbr.2012.04.048

[25] Fischl B, Dale AM (2000) Measuring the thickness of the human cerebral cortex from magnetic resonance images. Proceedings of the National Academy of Sciences 97, 11050–11055 10.1073/pnas.200033797 PMC27146 10984517

[26] Wierenga LM, Langen M, Oranje B, Durston S (2014) Unique developmental trajectories of cortical thickness and surface area. NeuroImage 87:120–126 24246495 10.1016/j.neuroimage.2013.11.010

[27] Winkler AM (2010) Cortical thickness or grey matter volume? The importance of selecting the phenotype for imaging genetics studies. NeuroImage 53:1135–1146 20006715 10.1016/j.neuroimage.2009.12.028 PMC2891595

[28] Jha SC (2019) Environmental influences on infant cortical thickness and surface area. Cereb Cortex 29:1139–1149 29420697 10.1093/cercor/bhy020 PMC6373689

[29] Panizzon MS (2009) Distinct genetic influences on cortical surface area and cortical thickness. Cereb Cortex 19:2728–2735 19299253 10.1093/cercor/bhp026 PMC2758684

[30] Kim P, Dufford AJ, Tribble RC (2018) Cortical thickness variation of the maternal brain in the first 6 months postpartum: associations with parental self-efficacy. Brain Struct Function 223:3267–3277 10.1007/s00429-018-1688-z PMC6358213 29855765

[31] Martínez-García M, Paternina-Die M, Desco M, Vilarroya O, Carmona S (2021) Characterizing the brain structural adaptations across the motherhood transition. Front global women’s health 2:742775 10.3389/fgwh.2021.742775 PMC8593951 34816246

[32] Oatridge A (2002) Change in brain size during and after pregnancy: study in healthy women and women with preeclampsia. Am J Neuroradiol 23:19–26 11827871 PMC7975506

[33] Luders E (2020) From baby brain to mommy brain: Widespread gray matter gain after giving birth. Cortex 126:334–342 32105976 10.1016/j.cortex.2019.12.029

[34] Luders E (2021) Gray matter increases within subregions of the hippocampal complex after pregnancy. Brain imaging Behav, 1–5 10.1007/s11682-021-00463-2 33881733

[35] Martínez-García M (2021) Do pregnancy-induced brain changes reverse? The brain of a mother six years after parturition. Brain Sci 11:168 33525512 10.3390/brainsci11020168 PMC7912216

[36] Lisofsky N, Gallinat J, Lindenberger U, Kühn S (2019) Postpartal neural plasticity of the maternal brain: Early renormalization of pregnancy-related decreases? Neurosignals 27:12–24 31112016 10.33594/000000105

[37] Luders E (2021) Signi cant increases of the amygdala between immediate and late postpartum: Pronounced effects within the superficial subregion. J Neurosci Res 99:2261–2270 34101893 10.1002/jnr.24855

[38] Mir FR, Pollano A, Rivarola MA (2022) Animal models of postpartum depression revisited. Psychoneuroendocrinology 136:105590 34839082 10.1016/j.psyneuen.2021.105590

[39] Cox JL, Murray D, Chapman G (1993) A controlled study of the onset, duration and prevalence of postnatal depression. Br J Psychiatry 163:27–31 8353695 10.1192/bjp.163.1.27

[40] Cardoso C, Fonseca A (2023) Mothers at-risk for postpartum depression: Mental health and emotion regulation throughout the postpartum period. Curr Psychol 42:12988–13002

[41] Marques R, Monteiro F, Canavarro MC, Fonseca A (2018) The role of emotion regulation difficulties in the relationship between attachment representations and depressive and anxiety symptoms in the postpartum period. J Affect Disord 238:39–46 29859386 10.1016/j.jad.2018.05.013

[42] Brummelte S, Galea LA (2016) Postpartum depression: Etiology, treatment and consequences for maternal care. Horm Behav 77:153–166 26319224 10.1016/j.yhbeh.2015.08.008

[43] Bernard K, Nissim G, Vaccaro S, Harris JL, Lindhiem O (2018) Association between maternal depression and maternal sensitivity from birth to 12 months: A meta-analysis. Attach Hum Dev 20:578–599 29374991 10.1080/14616734.2018.1430839

[44] Billot B (2020) Automated segmentation of the hypothalamus and associated subunits in brain MRI. NeuroImage 223:117287 32853816 10.1016/j.neuroimage.2020.117287 PMC8417769

[45] Fischl B (2002) Whole brain segmentation: automated labeling of neuroanatomical structures in the human brain. Neuron 33:341–355 11832223 10.1016/s0896-6273(02)00569-x

[46] Meltzer-Brody S, Rubinow D (2021) An overview of perinatal mood and anxiety disorders: epidemiology and etiology. Women’s Mood Disorders: A Clinician’s Guide to Perinatal Psychiatry, 5–16

[47] Shorey S (2018) Prevalence and incidence of postpartum depression among healthy mothers: A systematic review and meta-analysis. J Psychiatr Res 104:235–248 30114665 10.1016/j.jpsychires.2018.08.001

[48] Dennis C-L, Falah-Hassani K, Shiri R (2017) Prevalence of antenatal and postnatal anxiety: systematic review and meta-analysis. Br J Psychiatry 210:315–323 28302701 10.1192/bjp.bp.116.187179

[49] Murray L, Carothers AD (1990) The validation of the Edinburgh Post-natal Depression Scale on a community sample. Br J Psychiatry 157:288–290 2224383 10.1192/bjp.157.2.288

[50] El-Hachem C (2014) (biomedcentral. 10.1186/s12888-014-0242-7

[51] de Lange A-MG (2019) Population-based neuroimaging reveals traces of childbirth in the maternal brain. Proceedings of the National Academy of Sciences 116, 22341–22346 10.1073/pnas.1910666116 PMC6825266 31615888

[52] de Lange AMG (2020) The maternal brain: Region-specific patterns of brain aging are traceable decades after childbirth. Hum Brain Mapp 41:4718–4729 32767637 10.1002/hbm.25152 PMC7555081

[53] Blumenthal JD, Zijdenbos A, Molloy E, Giedd JN (2002) Motion artifact in magnetic resonance imaging: implications for automated analysis. NeuroImage 16:89–92 11969320 10.1006/nimg.2002.1076

[54] Esteban O (2017) Advancing the automatic prediction of image quality in MRI from unseen sites. PloS one 12 MRIQC:e0184661 10.1371/journal.pone.0184661 PMC5612458 28945803

[55] Ganzetti M, Wenderoth N, Mantini D (2016) Intensity inhomogeneity correction of structural MR images: a data-driven approach to define input algorithm parameters. Front neuroinformatics 10:10 10.3389/fninf.2016.00010 PMC4791378 27014050

[56] Magnotta VA, Friedman L, BIRN F (2006) Measurement of signal-to-noise and contrast-to-noise in the fBIRN multicenter imaging study. J Digit Imaging 19:140–147 16598643 10.1007/s10278-006-0264-x PMC3045184

[57] Esteban O, Markiewicz C, Blair R, Poldrack R, Gorgolewski K (2021) (Zenodo

[58] Gorgolewski K (2011) Nipype: a flexible, lightweight and extensible neuroimaging data processing framework in python. Front neuroinformatics 5:13 10.3389/fninf.2011.00013 PMC3159964 21897815

[59] Tustison NJ (2010) N4ITK: improved N3 bias correction. IEEE Trans Med Imaging 29:1310–1320 20378467 10.1109/TMI.2010.2046908 PMC3071855

[60] Avants BB, Tustison N, Song G (2009) Advanced normalization tools (ANTS). Insight j 2:1–35

[61] Zhang Y, Brady M, Smith S (2001) Segmentation of brain MR images through a hidden Markov random field model and the expectation-maximization algorithm. IEEE Trans Med Imaging 20:45–57 11293691 10.1109/42.906424

[62] Dale AM, Fischl B, Sereno MI (1999) Cortical surface-based analysis: I. Segmentation and surface reconstruction. NeuroImage 9:179–194 9931268 10.1006/nimg.1998.0395

[63] Klein A (2017) Mindboggling morphometry of human brains. PLoS Comput Biol 13:e1005350 28231282 10.1371/journal.pcbi.1005350 PMC5322885

[64] Raamana PR (2020) Visual QC Protocol for FreeSurfer cortical parcellations from anatomical MRI. BioRxiv, 2009. 2007.286807 (2020)

[65] Reuter M, Schmansky NJ, Rosas HD, Fischl B (2012) Within-subject template estimation for unbiased longitudinal image analysis. NeuroImage 61:1402–1418 22430496 10.1016/j.neuroimage.2012.02.084 PMC3389460

[66] Bates D (2015) Package ‘lme4’. convergence 12, 2

[67] Bates DM (2010) (Springer

[68] Kuznetsova A, Brockhoff PB, Christensen RH (2017) B. lmerTest package: tests in linear mixed effects models. J Stat Softw 82

[69] Kuznetsova A, Brockhoff PB, Christensen R (2015) H. B. Package ‘lmertest’. R package version 2:734

[70] Luke SG (2017) Evaluating significance in linear mixed-effects models in R. Behav Res Methods 49:1494–1502 27620283 10.3758/s13428-016-0809-y

[71] McCabe-Beane JE, Segre LS, Perkhounkova Y, Stuart S, O’Hara MW (2016) The identification of severity ranges for the Edinburgh Postnatal Depression Scale. J Reproductive Infant Psychol 34:293–303

[72] Seo S (2006) A review and comparison of methods for detecting outliers in univariate data sets. University of Pittsburgh

[73] Grande LA (2021) Postpartum stress and neural regulation of emotion among first-time mothers. Cogn Affect Behav Neurosci 21:1066–1082 34128217 10.3758/s13415-021-00914-9 PMC8565500

[74] Leuner B, Gould E (2010) Dendritic growth in medial prefrontal cortex and cognitive flexibility are enhanced during the postpartum period. J Neurosci 30:13499–13503 20926675 10.1523/JNEUROSCI.3388-10.2010 PMC2963448

[75] Strathearn L, Kim S (2013) Mothers’ amygdala response to positive or negative infant affect is modulated by personal relevance. Front NeuroSci 7:61638 10.3389/fnins.2013.00176 PMC3792358 24115918

[76] Barrett J (2012) Maternal affect and quality of parenting experiences are related to amygdala response to infant faces. Soc Neurosci 7:252–268 21943083 10.1080/17470919.2011.609907

[77] Rasia-Filho A, Fabian C, Rigoti K, Achaval M (2004) Influence of sex, estrous cycle and motherhood on dendritic spine density in the rat medial amygdala revealed by the Golgi method. Neuroscience 126:839–847 15207319 10.1016/j.neuroscience.2004.04.009

[78] Leckman JF, Herman AE (2002) Maternal behavior and developmental psychopathology. Biol Psychiatry 51:27–43 11801229 10.1016/s0006-3223(01)01277-x

[79] Numan M, Rosenblatt JS, Komisaruk BR (1977) Medial preoptic area and onset of maternal behavior in the rat. J Comp physiological Psychol 91:146 10.1037/h0077304 402400

[80] Numan M (1974) Medial preoptic area and maternal behavior in the female rat. J Comp physiological Psychol 87:746 10.1037/h0036974 4426995

[81] Darnaudéry M (2007) Early motherhood in rats is associated with a modification of hippocampal function. Psychoneuroendocrinology 32:803–812 17640823 10.1016/j.psyneuen.2007.05.012

[82] Leuner B, Mirescu C, Noiman L, Gould E (2007) Maternal experience inhibits the production of immature neurons in the hippocampus during the postpartum period through elevations in adrenal steroids. Hippocampus 17:434–442 17397044 10.1002/hipo.20278

[83] Pawluski JL, Galea LA (2006) Hippocampal morphology is differentially affected by reproductive experience in the mother. J Neurobiol 66:71–81 16216005 10.1002/neu.20194

[84] Schnakenberg P (2021) Examining early structural and functional brain alterations in postpartum depression through multimodal neuroimaging. Sci Rep 11:13551 34193913 10.1038/s41598-021-92882-w PMC8245412

[85] Deligiannidis KM (2013) GABAergic neuroactive steroids and resting-state functional connectivity in postpartum depression: a preliminary study. J Psychiatr Res 47:816–828 23499388 10.1016/j.jpsychires.2013.02.010 PMC3983790

[86] Parekh P (2024) Fast and efficient mixed-effects algorithm for large sample whole‐brain imaging data. Rep No FEMA:1065–9471 (Wiley Online Library 10.1002/hbm.26579 PMC10823765 38339910

[87] Pritschet L, Taylor CM, Santander T, Jacobs EG (2021) Applying dense-sampling methods to reveal dynamic endocrine modulation of the nervous system. Curr Opin Behav Sci 40:72–78 35369044 10.1016/j.cobeha.2021.01.012 PMC8975130

[88] Taylor CM, Pritschet L, Jacobs EG (2021) The scientific body of knowledge–Whose body does it serve? A spotlight on oral contraceptives and women’s health factors in neuroimaging. Front Neuroendocr 60:100874 10.1016/j.yfrne.2020.100874 PMC7882021 33002517

[89] Pritschet L (2020) Functional reorganization of brain networks across the human menstrual cycle. NeuroImage 220:117091 32621974 10.1016/j.neuroimage.2020.117091

[90] Taylor CM (2020) Progesterone shapes medial temporal lobe volume across the human menstrual cycle. NeuroImage 220:117125 32634592 10.1016/j.neuroimage.2020.117125

